# Evaluation of EGFR and RTK Signaling in the Electrotaxis of Lung Adenocarcinoma Cells under Direct-Current Electric Field Stimulation

**DOI:** 10.1371/journal.pone.0073418

**Published:** 2013-08-09

**Authors:** Hsieh-Fu Tsai, Ching-Wen Huang, Hui-Fang Chang, Jeremy J. W. Chen, Chau-Hwang Lee, Ji-Yen Cheng

**Affiliations:** 1 Institute of Biophotonics, National Yang-Ming University, Taipei, Taiwan; 2 Research Center for Applied Sciences, Academia Sinica, Taipei, Taiwan; 3 Biophotonics & Molecular Imaging Research Center, National Yang-Ming University, Taipei, Taiwan; 4 Institute of Biomedical Sciences, National Chung Hsing University, Taichung, Taiwan; 5 Department of Mechanical and Mechatronic Engineering, National Taiwan Ocean University, Keelung, Taiwan; University of Illinois at Chicago, United States of America

## Abstract

Physiological electric field (EF) plays a pivotal role in tissue development and regeneration. *In vitro*, cells under direct-current electric field (dcEF) stimulation may demonstrate directional migration (electrotaxis) and long axis reorientation (electro-alignment). Although the biophysical models and biochemical signaling pathways behind cell electrotaxis have been investigated in numerous normal cells and cancer cells, the molecular signaling mechanisms in CL1 lung adenocarcinoma cells have not been identified. Two subclones of CL1 cells, the low invasive CL1-0 cells and the highly invasive CL 1-5 cells, were investigated in the present study. CL1-0 cells are non-electrotactic while the CL 1-5 cells are anodally electrotactic and have high expression level of epidermal growth factor receptor (EGFR), in this study, we investigated the generally accepted hypothesis of receptor tyrosine kinase (RTK) activation in the two cell lines under dcEF stimulation. Erbitux, a therapeutic drug containing an anti-EGFR monoclonal antibody, cetuximab, was used to investigate the EGFR signaling in the electrotaxis of CL 1-5 cells. To investigate RTK phosphorylation and intracellular signaling in the CL1 cells, large amount of cellular proteins were collected in an airtight dcEF stimulation device, which has advantages of large culture area, uniform EF distribution, easy operation, easy cell collection, no contamination, and no medium evaporation. Commercial antibody arrays and Western blotting were used to study the phosphorylation profiles of major proteins in CL1 cells under dcEF stimulation. We found that electrotaxis of CL 1-5 cells is serum independent and EGFR independent. Moreover, the phosphorylation of Akt and S6 ribosomal protein (rpS6) in dcEF-stimulated CL1 cells are different from that in EGF-stimulated cells. This result suggests that CL1 cells’ response to dcEF stimulation is not through EGFR-triggered pathways. The new large-scale dcEF stimulation device developed in the present work will aid the sample preparation for protein-based experiments.

## Introduction

Aside from the commonly known chemical guidance, the physiological electric field (EF) established by transepithelial potential difference in tissues is also a directional cue for cell migration. The physiological EF participates in embryo development, morphogenesis, wound healing, neurogenesis, and neuro-regeneration as a directional cue and a morphogenetic field cue [1–6]. Many types of adherent cells, normal as well as cancerous, demonstrate directional migration (i.e., electrotaxis or galvanotaxis) and long axis reorientation (i.e., electro-alignment) under an *in vitro* direct-current electric field (dcEF) mimicking the physiological EF [7–9].

Several molecular mechanisms are involved in cell electrotaxis. Firstly, membrane potential depolarization by dcEF could directly activate voltage-gated ion channels or voltage sensitive proteins [10–13]. Secondly, one of the earliest observation of dcEF-cell interaction was cellular membrane components showing asymmetrical distribution under dcEF [14]. Biophysically, the membrane components are driven in combination of *in situ* electrophoresis [15,16], which is the lateral movement of charged components on the membrane driven by the dcEF, and electro-osmosis [17], in which charged membrane components were swept by electro-osmotic flow generated by the dcEF. Activation of asymmetrically distributed membrane components would lead to polarized cellular signaling which conveys the directional cue [18]. Biochemically, various membrane components perturbed under dcEF were involved in the electrotaxis of different cell types. The membrane components can be divided into four categories, membrane receptors, ion channels, receptor tyrosine kinases, and integrins. The intracellular signaling cascades reported in electrotaxis include PI3K, cAMP, PTEN, ERK1/2, and calcium signaling [11,19–23].

Being able to direct cancer cell migration, dcEF of physiological strength has been hypothesized to participate in cancer metastasis [10]. The electrotaxis of prostate cancer cells, lung adenocarcinoma cells, breast cancer cells, oral squamous cell carcinoma, and cervical carcinoma cells have been reported [10,24–29]. Voltage-gated sodium channel has been firstly reported to be involved in the electrotaxis of prostate cancer cells [10]. The electrotaxis of A549 lung adenocarcinoma cells and MDA-MB-231 breast cancer cells are shown to involve the epidermal growth factor receptor (EGFR) pathway [24,25]. Recently, the electrotaxis of HeLa cells, a cervical carcinoma cells, is shown to be dependent on a serine/threonine phosphatase and its substrate [29].

Lung cancer is the leading cause of cancer-related death in Taiwan and worldwide. We have been studying the CL1 lung adenocarcinoma cell line, which is derived from a patient with poorly differentiated lung adenocarcinoma. CL 1-5 and CL1-0 cells are subclones generated from CL1 cells by *in vitro* invasion assay. CL 1-5 cells have higher invasiveness and demonstrate anodal electrotaxis while CL1-0 cells have low invasiveness and demonstrate low electrotactic activity [26,30,31]. The CL 1-5 cells have high EGFR expression, similar to that in A549 cells and in MDA-MB-231 cells. However under dcEF stimulation, the EGFR on the CL 1-5 cells accumulates on the cathodal side while the cells migrate toward the opposite (anodal) direction [32]. In previous studies, the electrotaxis of the CL 1-5 cells was investigated in serum-free medium to exclude the influence from electro-migration of serum proteins [26,28,33].

In the present study, we investigated the involvement of serum and EGFR in the electrotaxis of CL 1-5 cells.

Erbitux is an intravenous therapeutic drug containing anti-EGFR monoclonal antibody, Cetuximab [34]. Erbitux binds to EGFR and prevents further binding to EGF and downstream activation of the receptor. Erbitux has been shown to inhibit tumor angiogenesis, invasion, and metastasis as well as cancer cell motility, proliferation, and survival. The drug’s therapeutic potential against non-small cell lung cancer is under investigation [35]. Erbitux has already been shown to inhibit the electrotaxis of A549 lung adenocarcinoma cells [25]. In the present study, a dual-field chip that allows the control of concurrent stimulations by EGF and dcEF, was developed and used for investigating the effect of Erbitux on the electrotaxis of CL 1-5 cells. An EGF stimulation following Erbitux incubation was used to verify the blocking efficacy of Erbitux.

EGFR is a member of the receptor tyrosine kinases, and many other RTKs have been reported to involve in the electrotaxis of different cells [36–40]. We extend the investigation of RTKs and intracellular signaling of CL1 cells under dcEF stimulation using a commercial array kit, PathScan RTK array kit, which screens for the activation of 28 RTKs and 11 intracellular signaling proteins. The array kit allows the recognition of specific phosphorylation sites (amino acid residues) related to the activations of the RTKs and the signaling proteins.

The amount of sample is crucial for biochemical analysis of phosphorylated proteins. In conventional dish-based devices for electrotaxis, coverslips were used to enclose the microfluidic chamber with a small culture area (<10 cm^2^) and thin cross section for a uniform EF stimulation [41,42]. Although these devices are suitable for cell migration study by light microscopy, the cell yields are usually low. A device with large culture area has been reported previously [43]. However, the device has non-uniform EF stimulation in the culture well and is only suitable for alternative-current (AC) applications. It also needs to be operated in a culture chamber to have contamination-free environment. In most of the conventional devices, to collect 5×10^6^ cells used for Western blotting as in previous studies [21], more than 4 batches of experiment are required for obtaining sufficient amount of sample. This introduces batch-to-batch and biological variations. Furthermore, in conventional devices, the cell culture regions are permanently sealed so cells cannot be collected efficiently without vigorous enzymatic treatment, which should be avoided for protein and protein phosphorylation analysis.

In our previous work [33], a large electric field chip (LEFC) was developed to collect mRNA for the gene expression analysis of CL 1-5 cells using microarray. Although LEFC provides uniform dcEF, the design could not be directly scaled up to a larger device because EF perturbation would occur. In addition, the LEFC was sealed and cannot be opened after experiments. Viable cells could not be easily recovered in LEFC without subjecting the cells to trypsin treatment and vigorous shearing, thereby limiting the applications of the chip.

To improve the cell yield and allow versatile applications, we designed a new PMMA chip called (e**X**tra **L**arge **E**lectric **F**ield **C**hip, XLEFC), which can provide uniform dcEF stimulation to cells in a large culture area. XLEFC can be quickly and easily disassembled after the experiment for viable cell collection. The phosphorylation dynamics of several RTKs and intracellular signaling proteins of CL1 cells under dcEF stimulation were explored. The protein phosphorylation dynamics between dcEF-stimulated CL1 cells and EGF-stimulated CL1 cells were compared to study the cellular signaling of CL1 cells under the two stimuli.

## Materials and Methods

### A: Cell culture and maintenance

The lung adenocarcinoma cell lines CL1-0 and CL 1-5 were acquired from Dr. Pan-Chyr Yang, National Taiwan University Hospital. *In vitro* transwell invasion assay has been performed on a clinical sample, CL1 tissue, to yield CL 1-5 cells, which has high invasiveness and anodal electrotaxis, and CL1-0 cells, which has low invasiveness and shows no electrotaxis [26,27,30,31].

Both cell lines were maintained in Dulbecco’s modified Eagle medium (DMEM, Life Technologies, USA) supplemented with 10% fetal bovine serum (FBS, Life Technologies, USA) under 37° C moist atmosphere with 5% CO_2_. The cells were passaged every 3 to 4 days. The cells used in the present work were within 10 to 20 passages. The cultured cells were mycoplasma-free as tested routinely every two weeks (e-Myco plus, iNtRON Biotech, Korea).

### B: Electrotaxis of CL 1-5 cells in serum-containing medium

The electrotaxis of the CL 1-5 cells in serum-free medium has been reported in previous studies [26]. The CL 1-5 cells showed anodal electrotaxis while the CL1-0 cells showed no electrotaxis. To verify the involvement of serum in the electrotaxis of the CL-1-5 cells, the electrotaxis experiment was carried out in medium containing 10% FBS using the MFUF chip (*m*ultiple-electric-*f*ield chip with *u*niform *f*low field) as previously reported [27]. Briefly, in MFUF chip, cell electrotaxis in three electric field strengths (EFS) and in the control condition (no electric field) are studied simultaneously in a single chip.

The migration trajectories of the CL 1-5 cells under dcEF (EFS = 300 mV/mm) for 2 hours were individually outlined and tracked by time lapsed imaging using a phase contrast microscope. The experiments were done in triplicate. Images were taken every 20 minutes. The data was plotted using the Ibidi chemotaxis and migration tool [44]. The definition of directedness and speed have been described in detail in the previous report [27]. Briefly, directedness is defined as average cosine of the angle included by the Euclidean distance vector and the EF vector. A cell migrating toward the cathode holds a directedness of +1 while a cell migrating toward anode holds -1. The directedness of a group of randomly migrating cells holds a value close to 0. All data was expressed as mean with errors of 95% confidence interval (CI).

### C: A dual-field chip for CL 1-5 cell electrotaxis study in both EF and chemical stimulation

**Figure 1:**
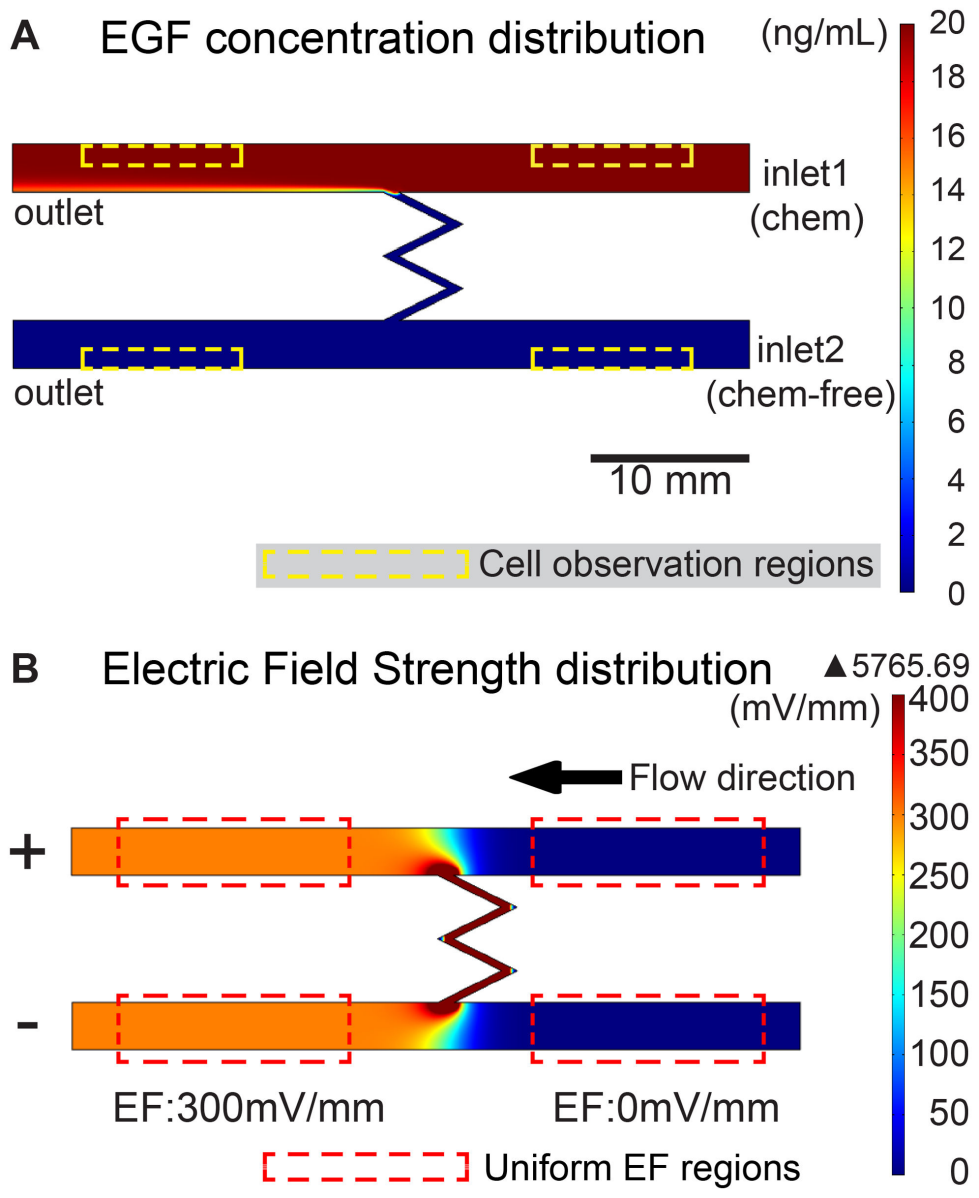
Simulated chemical concentration and electric field distribution of the dual-field chip. (A) The geometry and EGF concentration distribution in the dual field chip. Note that even for a small protein like EGF, only a small portion of it would be transported and create localized chemical gradient. Regions for cell observation are outlined in yellow dash lines. (B) The dcEF strength distribution in the dual field chip. Note that cell electrotaxis and random migration (control) with and without EF can be studied in a single chip. Regions with uniform EF distribution are outlined in red dash lines.

A dual-field chip was a slight modification from one of our previous works, MFUF chip [27]. The dimension of this dual-field chip is similar to that of MFUF chip. A dual-field chip has two inlets and two outlets (Figure 1A). Two 3 mm-wide channels were connected at the middle with a narrow zigzag channel (width 0.5 mm) for conducting electric current and for limiting chemical transportation. The media with and without the chemical were infused separately from inlet 1 and inlet 2 respectively at a flow rate of 20 µL/hr. The electric current flew from one outlet to another. Uniform EF was obtained in the left parts of the two channels and no EF was present in the right parts of the two channels (Figure 1B). The medium flow direction was from the EF-null regions to EF regions and avoided the possible effect on the control cells from the cell products secreted by the dcEF-stimulated cells. By such a design, cell electrotaxis and random migration in two chemical conditions can be observed in the four regions in a single chip in a single experiment.

The *laminar flow*, *chemical species transport* and *dc/ac* simulations were carried out in COMSOL Multiphysics software (COMSOL Inc., USA). A 300 mV/mm EFS in the DMEM with conductivity of 1.38 S·m^-1^ [45] was simulated. The chemical diffusion and electrophoretic migration of 20 ng/mL epidermal growth factor (EGF) with a diffusion coefficient of 1.6x10^-10^ m^2^/s were simulated [46,47]. At low flow rate of 20µL/hr, due to the low molecular weight (6045 Da) and the electrophoresis of EGF, a slight concentration difference occurred near the zigzag channel. In our previous work, we have found that the diffusion simulation is in accordance with the experimental data [48]. Therefore, cells were observed in the regions with uniform chemical concentration and EF (Figure 1).

### D: EGFR and the electrotaxis of CL 1-5 cells

To investigate the involvement of EGFR pathway in the electrotaxis of CL 1-5 cells, dcEF stimulation with and without the presence of Erbitux [25] were applied in both a single field chip [26] and a dual-field chip.

In the single-field chip, the CL 1-5 cells were pretreated with the serum-free DMEM with or without 4 nM Erbitux for 16 hours on the chip. Afterward, the electrotaxis of the CL 1-5 cells in a 300 mV/mm EFS was observed. In the dual-field chip, 4 nM Erbitux in serum-free DMEM was infused from inlet 1 and the serum-free DMEM (no Erbitux) was infused from inlet 2. The electrotaxis under 300 mV/mm EFS and the random migration of the CL 1-5 cells in the serum-free medium with and without Erbitux were studied in the same chip simultaneously.

To verify that EGFR on the cells were effectively inhibited by the Erbitux, CL 1-5 cells were cultured in serum-free medium with and without 4 nM Erbitux for 16 hours and then stimulated with 20 ng/mL EGF (Sigma-Aldrich, USA).

The electrotaxis and the random migration of the CL 1-5 cells were observed for up to two hours. More than 100 cells from duplicate experiments were analyzed. Image and data analysis procedures were the same as those described in section B.

### E: XLEFC design and fabrication

**Figure 2:**
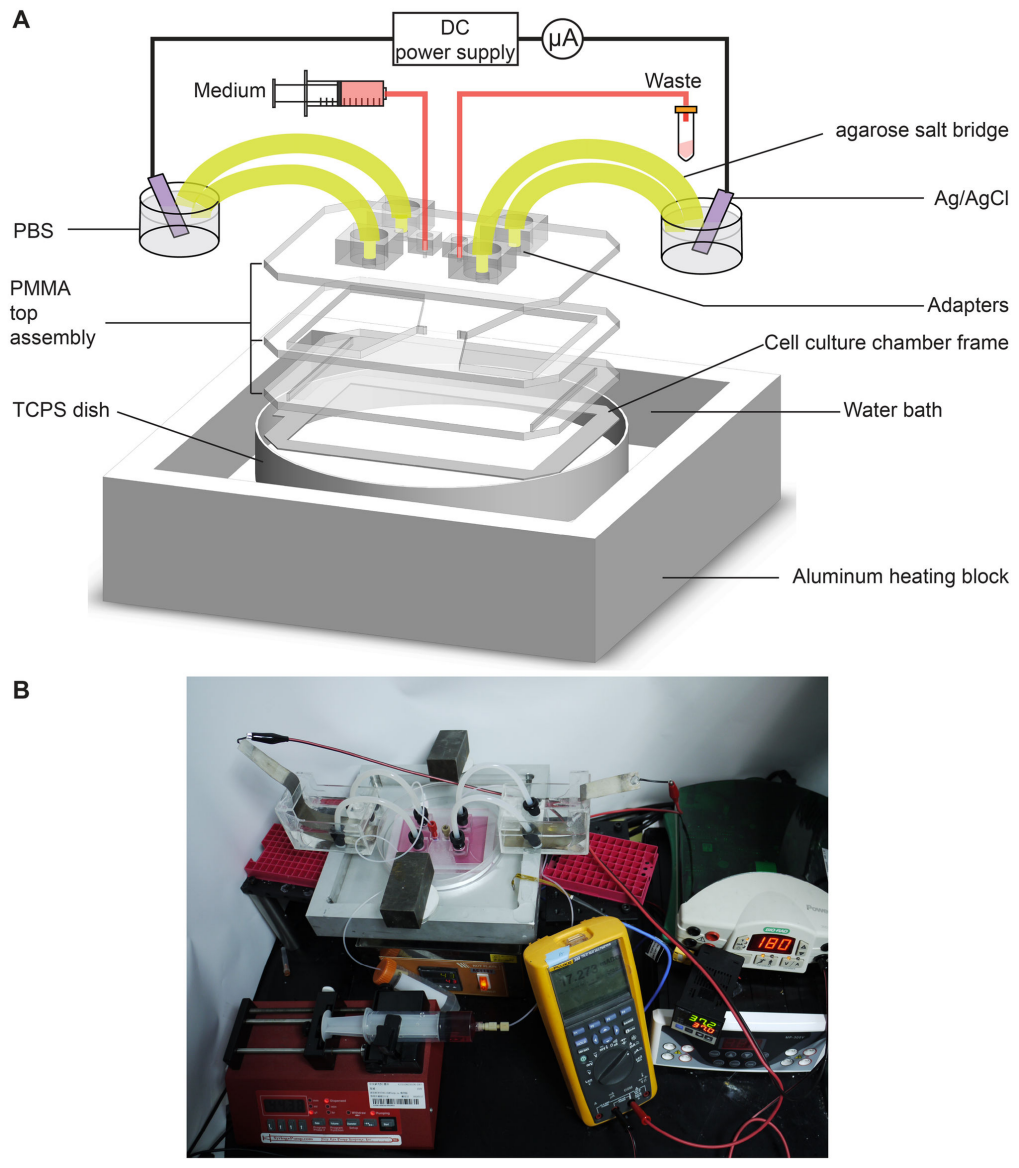
Setup of the electrotaxis experiment using XLEFC. (A) XLEFC is composed of the top assembly, the frame of the cell culture chamber, and a TCPS dish. Stable temperature is provided by water bath. Electric field stimulation is applied by a DC power supply through Ag/AgCl plate electrodes. (B) A photo picture showing the setup on a general laboratory bench.

XLEFC chip was composed of a PMMA top assembly, the frame of cell culture chamber, and the 150 mm TCPS dish (430599, Corning, USA) (Figure 2).

**Figure 3:**
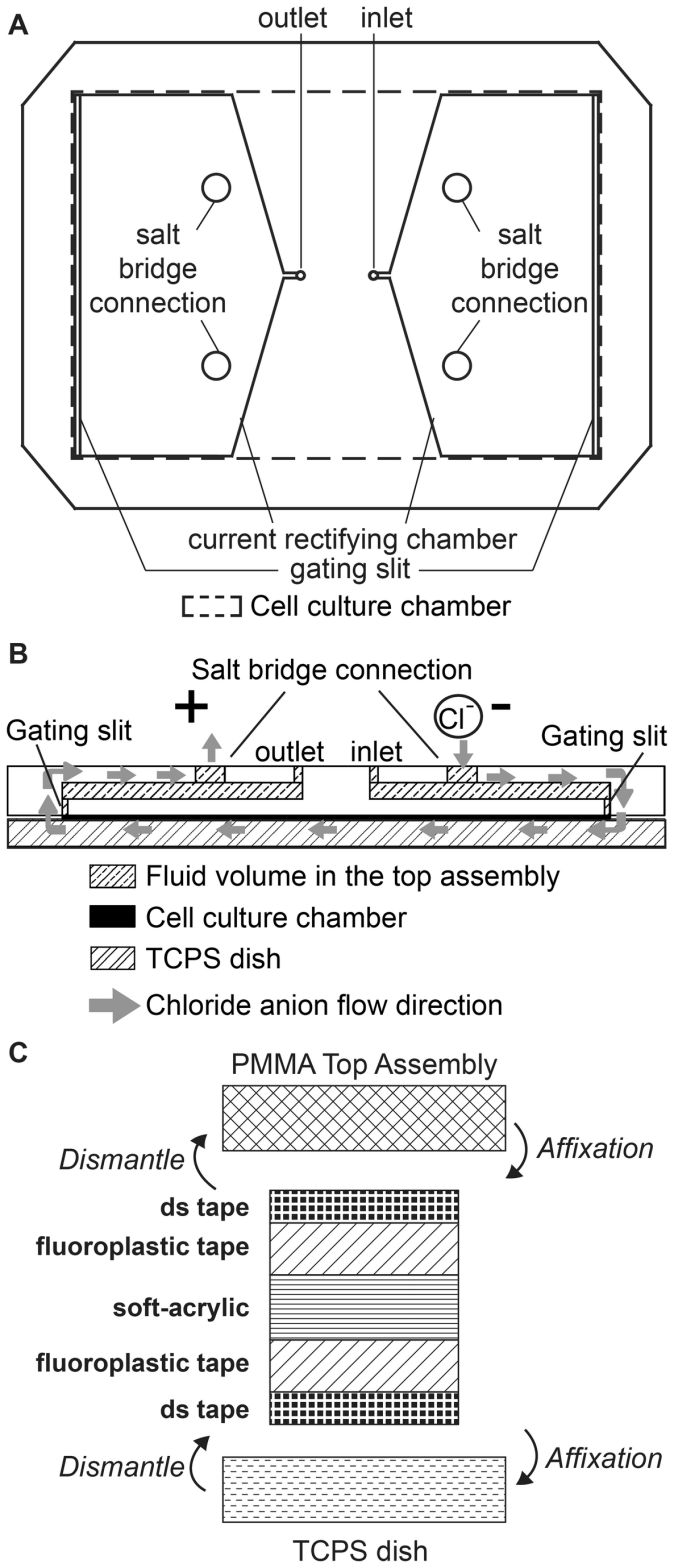
Detailed configuration of XLEFC. (A) The top view of the XLEFC top assembly design. The electrical current enters from the salt bridge connections through the current rectifying chamber and the gating slit, and then into the culture chamber. (B) The side view of XLEFC showing the integration of the top assembly, the cell culture chamber, and the TCPS dish. The flow direction of chloride ions, which are driven by the external dcEF, is shown in grey arrows. (C) The frame of the cell culture chamber has five layers, including one layer of soft-acrylic backbone, two layers of fluoroplastic tape, and two layers of double-sided tape (dstape). The design enables airtight seal during the experiment and quick release after the experiment.

The PMMA top assembly was composed of three layers of 3 mm PMMA plates. The top layer contained the medium inlet, the outlet and the connections for the salt bridges. The middle layer contained two current rectifying chambers (the two pentagons) where electric current could spread out from the salt bridges to the 1 mm-long and 69 mm-wide gating slits in the bottom layer (Figures 2 and 3). Microscopically, the electric current was carried by chloride ion flow. The chloride ions were generated by the electrolysis process at the cathode and then moved through the cathode-side salt bridge, the cell culture chamber and the anode-side salt bridge. The chloride ions then deposited on the anode as AgCl and closed the electric circuit. The large size of the current rectifying chamber allowed the chloride ions to disperse in the chambers when the current flew through the chambers. Ions then uniformly passed through the gating slit and travelled homogeneously across the cell culture chamber (Figure 3B). A uniformly distributed electric current would establish a uniform EF in the culture chamber, where most cells reside. The uniform EF is better visualized by a numerical simulation, as shown in section C in Results and Discussions. This design can be further adapted to create even larger bioreactors for uniform electric stimulation on larger number of cells.

The pattern for each PMMA layer were designed in AutoCAD (Autodesk, Inc., USA) and cut on a piece of 3 mm-thick PMMA plate by a CO_2_ laser scriber (ILS-II, LTT Corp., Taiwan). The three layers were thermally bonded and then adapters for the connections of the tube fittings were adhered to the top assembly by cyanoacrylate glue.

The frame of the cell culture chamber enclosing a cell culture area of 100 mm × 69 mm × 0.6 mm (L × W × H) was constructed by multiple layers of films including (1) one layer of 0.2 mm soft-acrylic sheet (CRD Inc., Japan) (2), one layer of 0.13 mm fluoroplastic tape (ASF-110 FR, Chukoh Chemicals, Japan) on both sides, and (3) one layer of 0.07 mm double-sided tape (PET8018, 3M, USA) on both fluoroplastic tapes. For affixation the double-sided tapes were adhered on the non-sticky face of the fluoroplastic tapes (Figure 3C).

The pattern of the cell culture chamber (Figure 3) was designed and cut by the same procedure as the PMMA layers but with lower laser power. The double-sided tape had limited adhesiveness to the non-sticky face of the fluoroplastic tape. This property allowed airtight sealing during the cell culturing experiment and quick detachment of the top assembly immediately after the experiment (Figure 3C). Viable cells could be recollected and cultured or washed thoroughly and lysed. The large cell culture area of XLEFC (69 cm^2^) allowed 3-7×10^6^ sub-confluent single layer of CL1 cells, corresponding to cell lysate containing 0.8-1 mg total protein to be isolated in a single device.

After the experiment, the cell culture chamber, with the double-sided tapes being removed, was disinfected in 1% Virkon S (DuPont Corp, USA) and reused after affixing new double-sided tapes, minimizing inter-device variation.

The advantages of XLEFC include simple device fabrication and assembly, easy cell seeding, airtight sealing, no evaporation and no contamination, uniform EF stimulation, low Joule heating, quick disassembly after experiment, and convenient viable cell collection.

### F: Numerical simulation of EF in XLEFC

A three-dimensional model of fluids in XLEFC was created in COMSOL Multiphysics software. An electric current stationary stimulation was done using the *dc/ac* module. Electric conductivity of 1.38 S·m^-1^ and relative permittivity of 80 was used for the liquid buffer in the simulation. The simulation result was visualized and outputted in COMSOL. The electric current *I* required to create a 300mV/mm EFS inside the cell culture chamber was calculated by Ohm’s law, *I*=*σEA*, where σ and *A* are electrical conductivity of the liquid buffer and the cross sectional area of the culture chamber, respectively. A 17.14 mA direct current flowing through the 69 mm x 100 mm x 0.6 mm cell culture chamber established a 300 mV/mm EFS.

### G: Electric field and temperature measurement

To measure the electric field and the temperature in XLEFC, the chip was slightly modified so that the rectifying chamber did not stack with the cell culture chamber. Opening holes with distance of 10 mm (diameter 0.3~0.5 mm) were drilled on the surface above the cell culture area on the top assembly using a CO_2_ laser scriber. These opening holes allow measurement of EFS by home-made Ag/AgCl wire electrodes [27] or temperature by a K-type thermocouple (TPK-01, Tecpel Inc., Taiwan).

The voltage drops (ΔV) between the Ag/AgCl electrodes inserted in two adjacent holes were measured by a digital multimeter (True-RMS 289, Fluke, USA). The EFS was calculated by dividing the measured ΔV by the distance between two holes. The temperature distributions in XLEFC before and after 2 hours of 300 mV/mm dcEF stimulation were measured by the same digital multimeter. All the data was plotted using a home-written MATLAB program.

### H: Electric field stimulation and cell lysis

The frame of the cell culture chamber was first affixed on TCPS dishes and disinfected for 20 minutes under UV light. For each XLEFC chip, lung adenocarcinoma cells of one 75T flask were trypsinized (0.25% trypsin-EDTA, Life Technologies, USA) and re-suspended in DMEM with 10% FBS before seeding into the chip.

Cell seeding in XLEFC is similar to that for routine cell culturing. The cell suspension was counted in a hemocytometer using trypan blue (Sigma-Aldrich, USA) and 3x10^6^ cells were seeded into the area enclosed by the frame of the cell culture chamber. The cells were allowed to adhere to the TCPS dish for 6 hours in a CO_2_ incubator.

Before the experiments, the sub-confluent monolayer cells were washed twice with 1X PBS and the PMMA top assembly was then affixed to the frame of the cell culture chamber by the double-sided tape on the top, enclosing the cell culture chamber and completing the assembly of XLEFC. The XLEFC was then filled with DMEM slowly and carefully to avoid entrapment of bubbles in the cell culture chamber.

The setup diagram and a photo picture are shown in Figure 2. Four 20 cm salt bridges (filled with 1.5% agar dissolved in 1X PBS) were connected to the XLEFC chip. The lower part of the TCPS dish was immersed into a 37° C water bath to provide stable temperature for cell culture and then a dcEF was applied by an electrophoresis power supply (PowerPac Basic, Bio-Rad, USA) through Ag/AgCl plate electrodes immersed in 1X PBS. The required current was monitored by a multimeter (true-rms 289, Fluke, USA) and controlled at 17.14±0.3 mA. The cells for control group were prepared in the same way except no electric potential was applied.

After electric field stimulation, XLEFC was chilled on ice and the PMMA top assembly was levered off. The cells on the TCPS dish were then washed three times with ice cold 1x PBS and lysed in cold lysis buffer (#9803, Cell Signaling Technology, USA) supplemented with 1mM phenylmethylsulfonyl fluoride (PMSF). Cells were incubated with the lysis buffer for 10 minutes and then centrifuged at 16100xg for 10min at 4° C (Centrifuge 5415R, Eppendorf, USA). The supernatant was stored at -80° C until use.

### I: PathScan^®^ RTK signaling antibody array kit experiment

The samples for the experiment were quantified by Bradford assay (Protein Assay, Bio-Rad, USA). 150µg of cell lysates prepared as previously described were incubated with the PathScan RTK signaling kit array chip (#7949, Cell Signaling Technology, USA) according to the protocol provided by the manufacturer. Briefly, after incubation of the samples, biotin-labeled anti-pan-phospho-tyrosine antibodies and specific anti-phospho-residue antibodies were used to detect phosphorylated proteins captured on each spot on the nitrocellulose membrane. After incubation with DyLight 680-linked streptavidin, the chip was washed, fully dried and imaged under a GeneTAC UC4x4 Microarray Analyzer (Genomic Solutions Inc., USA) using the excitation and emission filter set for Cy5 spectrum with a 695 nm band-pass filter.

The fluorescence spots were analyzed by densitometry using Fiji ImageJ [49] and Gilles Carpentier’s Protein Array Analyzer macro [50]. Experimental data from triplicate experiments were normalized against negative controls and expressed as mean ± SEM.

### J: Statistical Analysis

In the electrotaxis assay of the CL1 cells, statistical inferences between cell groups with different treatments were conducted by Student’s t test or one-way analysis of variance (ANOVA) with Tukey’s post-test using the commercial software Prism 5.0 (GraphPad Software, CA, USA). A difference between two groups with a P value smaller than 0.05 is considered to be significant.

In Pathscan RTK signaling antibody array experiment, two-way ANOVA using repeated measures was conducted with cell type and EF stimulation time as independent variables. Differences were further investigated using post-hoc Bonferroni test when the statistically significant ANOVA was indicated.

The asterisk (*) denotes *p*<0.05, the double asterisks (**) denotes *p*<0.01, and the triple asterisks (***) denotes *p*<0.001.

### K: SDS-PAGE and Western blotting

CL 1-5 and CL1-0 cells were stimulated by dcEF for different durations in serum-free medium or serum-containing medium in XLEFC and lysed with cold lysis buffer (#9803, Cell Signaling Technology, USA, supplemented with 1mM PMSF and 50mM NaF) and centrifuged for 10 minutes at 16100xg, 4° C. 4X loading buffer (250mM Tris-Cl pH6.8, 8% Sodium Dodecyl Sulfate (SDS), 40% glycerol, 20% β-mercaptoethanol, 0.04% bromophenol blue) were mixed with cell lysates and boiled for 10 minutes at 95° C.

50 µg total protein was loaded into 10% discontinuous SDS polyacrylamide gel for electrophoresis and then transferred onto polyvinylidene fluoride (PVDF) membranes. Membranes were blocked in 5% bovine serum albumin (BSA, Sigma, USA) and incubated with primary antibodies overnight at 4° C (anti-Akt antibodies were from Cell Signaling Technology and all others were from Abcam, UK) in Tris buffered saline with 0.1% Tween 20 (TBST). After washing in TBST, membranes were incubated in horseradish peroxidase-conjugated antibodies (Abcam, UK) for 1 hour at room temperature.

Membranes were then incubated with chemiluminescence reagent VisGlow (Visual Protein Biotechnology Corp, Taiwan). Western blotting images were taken under the Molecular Imager Chemidoc XRS+ (Bio-Rad, USA) and analyzed in Image Lab (Bio-Rad USA) or Fiji ImageJ. Experiments were done in triplicate. Under each condition of the Western blotting experiments, β-tubulin was used as the internal control and all densitometric values were normalized against the relative β-tubulin value.

### L: CL1 cell stimulation by EGF

CL1 cells were grown in 10 cm TCPS dishes to 80% confluence and then starved in serum-free medium for 16 hours. The cells were then conditioned with 20 ng/mL of EGF in serum-free DMEM for 30 minutes. Cells were washed twice with cold 1X PBS and harvested using a scraper. After that, cells were immediately lysed, denatured in denaturing Laemmli buffer, and subjected to SDS-PAGE and Western blotting analysis.

## Results and Discussion

### A: CL 1-5 electrotaxis is serum independent

To avoid the influence of possible chemical concentration gradient generated from electro-migrating serum proteins, the electrotaxis of CL 1-5 cells has been studied in serum-free medium [26]. Unlike several primary cells, in which serum-deprivation abolishes electrotaxis [41,51], CL 1-5 cells demonstrate anodal electrotaxis in serum-free medium.

**Figure 4:**
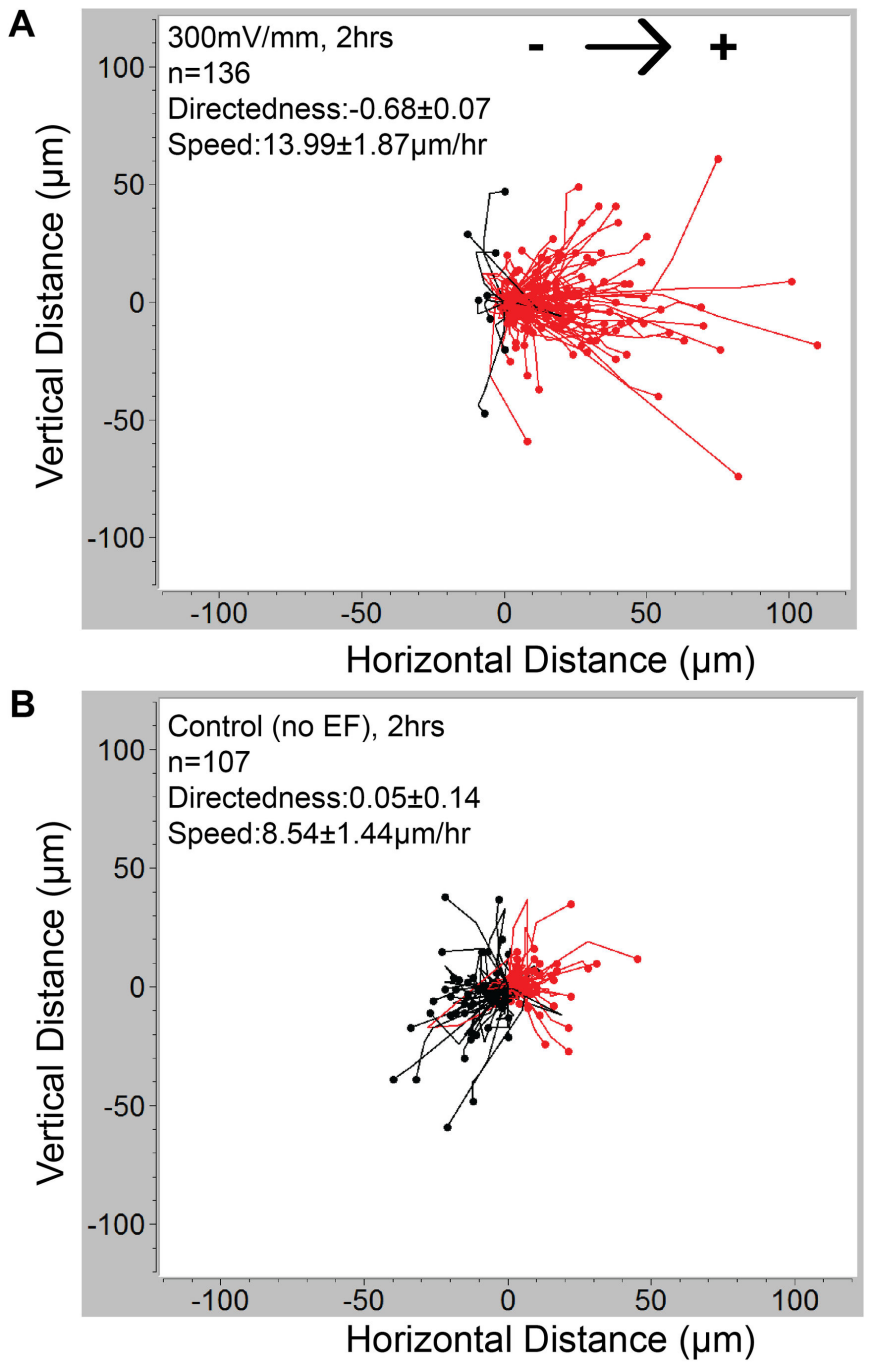
The migration trajectories of CL 1-5 cells for 2 hours in serum-containing medium. (A) CL 1-5 under 300mV/mm dcEF shows anodal electrotaxis. Cell tracks with end positions to the right appear in red and those to the left appear in black. (B) The random migration of CL 1-5 cells without dcEF stimulation.

In this study, we first investigated the electrotaxis of CL 1-5 cells in the serum-free and the serum-containing medium in the MFUF chip [27]. The electrotaxis migration trajectories of the CL 1-5 cells in the serum-containing medium under a 300 mV/mm EFS and the random migration trajectories in the control experiment for 2 hours were shown in Figure 4. The CL 1-5 cells in the serum-containing medium without dcEF stimulation exhibited a directedness of 0.05±0.14 while under dcEF, the directedness of CL 1-5 cell electrotaxis is -0.68±0.07 (*p*<0.0001). Also the cell migration speed was increased from 8.54±1.44 µm/hr to 13.99±1.87 µm/hr under electric field stimulation (*p*<0.0001). In comparison, in serum-free medium, the directedness was -0.06±0.14 without dcEF and -0.73±0.06 when under 300mV/mm EFS (*p*<0.0001). Detailed results of CL 1-5 electrotaxis in serum-containing and serum-free media are shown in Table 1.

**Table 1:** The directedness and speed of CL 1-5 electrotaxis in various conditions.

			**Directedness (Σcosθ/n)**			**Speed(µm/hr)**		
**Medium**	**EF(V/m)**	**N**	**Mean**	**s.e.m**	**SD**	**Mean**	**s.e.m**	**SD**
**Serum**	0	136	0.05	0.07	0.74	8.54	0.72	7.43
	300	107	-0.68	0.04	0.44	13.99	0.94	10.91
**Serum-Free**	0	107	-0.06	0.07	0.71	6.13	0.38	3.94
	300	233	-0.73	0.03	0.44	10.5	0.51	7.76
**4nM Erbitux**	0	134	0.00	0.06	0.71	6.37	0.38	4.38
	300	193	-0.71	0.03	0.46	10.15	0.52	7.26
**20ng/mL EGF**	0	133	0.26	0.05	0.62	12.29	1.03	11.9
	300	111	-0.51	0.06	0.58	12.55	0.96	10.15
**Erbitux then EGF**	0	133	-0.07	0.06	0.69	7.02	0.46	5.31
	300	120	-0.71	0.03	0.36	10.59	0.61	6.65

The directedness of the electrotaxis of the CL 1-5 cells was not affected whether the medium contained the serum or not (-0.73±0.06 versus -0.68±0.07, *p*=0.24). However, the CL 1-5 cells in the serum-containing medium migrated faster than those in the serum-free medium. Without dcEF stimulation, CL 1-5 cell migration speed in the serum-free medium was 6.13±0.76 µm/hr while in the serum-containing medium the speed increases to 8.54±1.44 µm/hr (*p*=0.0034). Under 300mV/mm dcEF stimulation, the speed of the CL 1-5 cells in the serum-containing medium was higher than that in the serum-free medium (13.99±1.87 versus 10.5±1.02 µm/hr, *p*=0.0004).

The results described above suggest that in both media, a 300 mV/mm dcEF not only guided the migration but also increased the migration speed to almost two folds compared to those in the control group. However, the serum in the medium is not necessarily involved in the directional decision in the electrotaxis of the CL 1-5 cells, but it merely accelerates cell migration. This acceleration could be due to activation of cells by the numerous growth factors contained in the serum [52].

### B: CL 1-5 electrotaxis is EGFR independent

In the electrotaxis of A549 lung adenocarcinoma cells, inhibition of EGFR signaling by Erbitux retards the electrotactic directedness from 0.76±0.12 to 0.53±0.08, indicating the participation of EGFR signaling [25]. Under dcEF stimulation, CL 1-5 cells show cathodal accumulation of EGFR although this accumulation occurs at the opposite direction of the anodal electrotaxis [32]. In the present study, the involvement of EGFR pathway in CL 1-5 cell electrotaxis was investigated using Erbitux.

Without dcEF stimulation, the directedness of Erbitux-treated CL 1-5 cells was the same as that for untreated cells (0.00±0.12 versus -0.06±0.14, *p*>0.05). The speed of Erbitux-treated CL 1-5 cells and untreated cells were also similar (6.37±0.76 versus 6.13±0.76 µm/hr, *p*>0.05). This result confirms that Erbitux treatment does not affect the random migration direction and the speed of CL 1-5 cells.

Under the dcEF stimulation, Erbitux-treated CL 1-5 cells showed anodal electrotaxis with directedness of -0.71±0.06 and speed of 10.15±1.06 µm/hr. Such behavior is almost identical to the electrotaxis of the CL 1-5 cells without Erbitux treatment (directedness of -0.73±0.06 and speed of 10.5±1.02 µm/hr, *p*>0.05). This result indicates that the blocking of EGFR by Erbitux does not inhibit the electrotaxis of the CL 1-5 cells.

The involvement of EGFR in CL 1-5 cell’s electrotaxis was further investigated by the following tests: The EGFR on the CL 1-5 cells is functional and the Erbitux is effective toward the EGFR on the cells.

**Figure 5:**
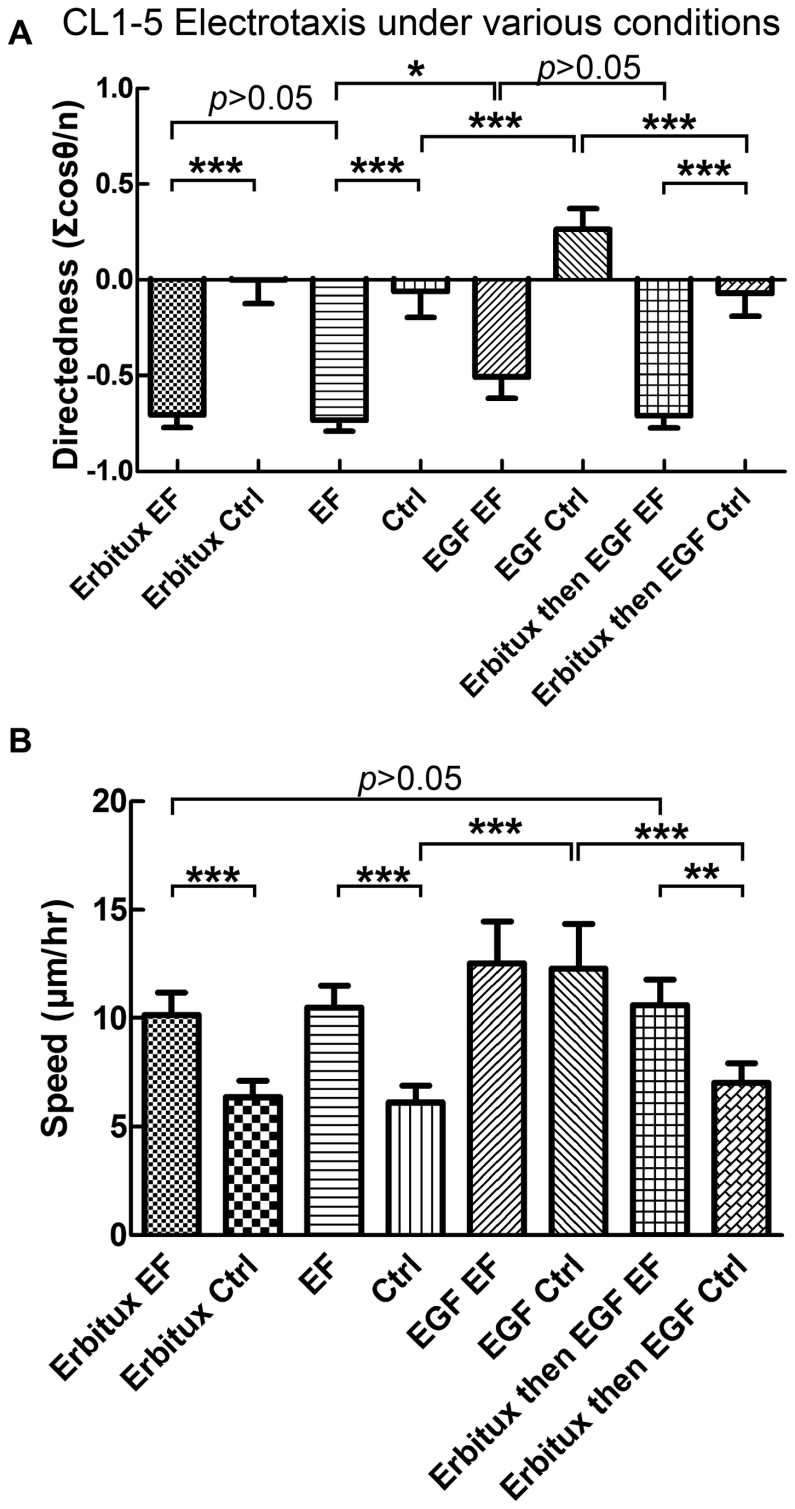
Electrotaxis of CL 1-5 cells under various conditions. (A) The directedness of CL 1-5 cell electrotaxis and random migration in medium with or without 4nM Erbitux and with or without EGF stimulations. (B) The speed of CL 1-5 cell electrotaxis and random migration in medium with or without 4nM Erbitux and with or without subsequent EGF stimulation.

Under EGF stimulation (no dcEF), CL 1-5 cells showed directional migration with directedness of 0.26±0.10 compared to the directedness of -0.06±0.14 in the control group (*p*<0.001) (Figure 5A). This directional migration might be due to the directional cue given at the time of EGF infusion in the dual-field chip. Under the EGF-only stimulation, the migration speed of CL 1-5 cells increased to 12.29±2.06 µm/hr in contrast to that of the control group with 6.13±0.76 µm/hr (*p*<0.001). The apparent difference indicates that CL 1-5 cells are indeed susceptible to EGF stimulation and that EGF increases the cell migration speed [53–55].

Under the simultaneous stimulation of EGF and dcEF (300mV/mm EFS), CL 1-5 cells showed anodal electrotaxis with directedness of -0.51±0.12 and speed of 12.55±1.92 µm/hr. A slight decrease in the directedness in comparison to the electrotaxis without the coexisting EGF was observed (*p*<0.05) although the migration speed is the same (*p*>0.05). The partial loss in the directedness may be caused by the polarized EGFR signaling owing to the accumulation of EGFR on the cathodal side of the cells [32] or the directional cue provided at the time of the EGF infusion in the dual-field chip. The similar migration speed for these two groups suggests that the EGF and the EF stimulation may elicit some shared intracellular signaling. Alternatively, the CL 1-5 cells’ migration machinery may have reached full activity by the EGF stimulation alone and cannot migrate faster under the existing dcEF stimulation.

The efficacy of Erbitux toward EGFR was verified by comparing the effect of the EGF stimulation on the CL 1-5 cells with and without Erbitux pretreatment. With EGF stimulation, the random migration speeds of treated and untreated cells were 7.02±0.92 µm/hr and 12.55±1.92 µm/hr, respectively. The effect of the Erbitux treatment was significant (*p*<0.001). This result indicates that Erbitux is indeed effective and the pretreatment protects the CL 1-5 cells from being stimulated and activated by the added EGF (Figure 5).

Overall, the above results indicate that firstly, EGF indeed activates CL 1-5 cells, secondly, Erbitux does exert blocking on the EGFR on the cells, and thirdly, blocking of EGFR does not affect the electrotaxis of CL 1-5 cells. Detailed result of the electrotaxis of the CL 1-5 cells with and without Erbitux treatment and EGF stimulation is shown in Table.1.

With only the EGF stimulation, the directedness of the random migration of the CL 1-5 cells was -0.26±0.10 and that of the Erbitux-treated CL 1-5 cells was -0.07±0.12. The difference was statistically significant (*p*<0.001) suggesting that EGF stimulation in our experiment elicited directional migration (Figure 5A and Table.1) The reason of this directional migration is not clear. One possibility is the directional cue given by the EGF infusion in the dual-field chip. In the dual-field chip experiment, the EGF was infused from inlet 1 in Figure 1. Therefore, at the beginning of the EGF infusion, the cells in the chip were initially subjected to a chemical gradient that has high EGF concentration at the right side of the chip. The corresponding gradient direction was opposite to the dcEF direction. The abolishment of the directedness (-0.07±0.12) of the Erbitux-treated CL 1-5 cells supported the involvement of the EGF gradient. Although the observation of the migration trajectories of the CL 1-5 cells were carried out after the EGF gradient had diminished, the initial directional cue may have exerted lasting effect on the cells. This interesting phenomenon is worth of further investigation.

To confirm that the electrotaxis of CL 1-5 cells is independent of EGFR and to further understand the underlying molecular signaling mechanism, protein expression and phosphorylation were analyzed and described in following sections. In the present work, the stimulation by EGF was conducted using constant concentration. A study investigating the directional migration of CL 1-5 cells under coexisting dcEF and EGF concentration gradient is currently underway to elucidate the inter-relationship between electrotaxis and chemotaxis.

### C: Uniform electric field stimulation is provided by the XLEFC chip

The analysis of protein expression and phosphorylation analysis requires that the cells cultured in XLEFC be homogeneously subjected to EF stimulation and culturing temperature. We first verified the performances of XLEFC.

**Figure 6:**
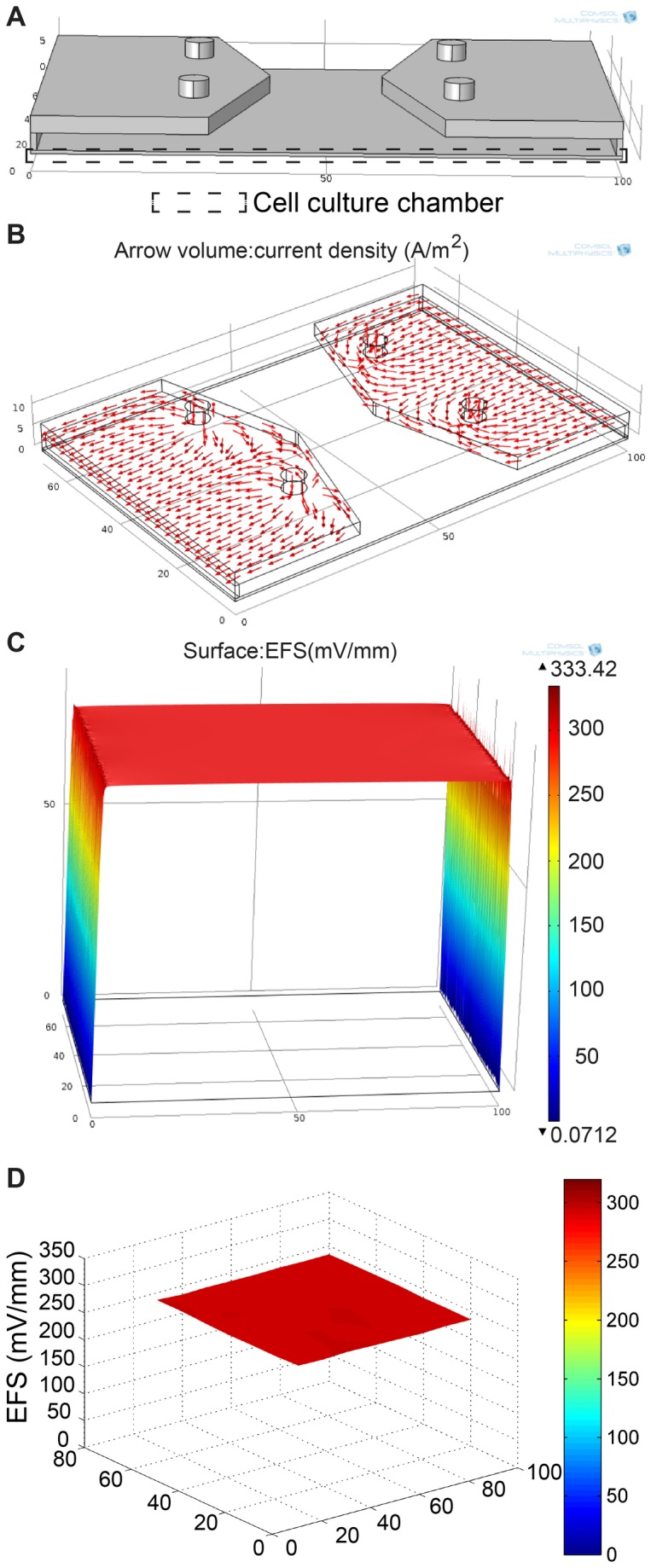
The homogeneous electric field distribution in XLEFC. (A) The 3D model of electrolyte-filled XLEFC built using COMSOL. (B) The vector volume plot shows the rectifying effect of electric current in the rectifying chamber. Uniform electric current is obtained at the gating slits. (C) The simulated EFS distribution at the bottom of the cell culture chamber shows extremely uniform dcEF in the cell culture chamber. (D) The plot of measured EFS taken in the cell culture chamber, excluding the gating slits.

A 3D model of electrolyte-filled XLEFC was built (Figure 6A) and the EF distribution in the chip was simulated. The current density (A/m^2^) in the current rectifying chamber is shown as vector volume in Figure 6B. The electric current flows into the culture chamber from the salt bridges. As seen in the simulated current vector, the current spreads out evenly in the rectifying chamber and then runs across the cross-section to the gating slit before it enters the culture chamber. Thus, a uniform electric field was obtained in the cell culture chamber (Figure 6C). The cells residing directly beneath the gating slits (1 mm x 69 mm on each side) may subject to slightly non-uniform EF. However, the number of cells in this region is negligible compared to that in the culture chamber (100 mm x 69 mm). From the simulation, the mean EFS obtained in the cell culture chamber at 5 µm (adherent cell height) was 299.6 mV/mm with coefficient of variation (CV) of 1.2%. Also, because the cells were seeded prior to mounting of the top assembly, there was no cell in the current rectifying chamber and the majority of the cells in the cell culture chamber were stimulated by the uniform EF.

We also measured the actual EFS in XLEFC by inserting Ag/AgCl wire electrodes into the cell culture chamber. The measured EFS in XLEFC had a mean of 281.3 mV/mm with standard deviation of 6.6 mV/mm (Figure 6D). The CV of the measured EFS was 2.3%. This showed that the XLEFC indeed provides uniform EF stimulation to the cells in the large area of the cell culture area. The design principle of XLEFC can be applied to create larger EF bioreactors. This will benefit biochemical and molecular biological research in cell-EF interaction. Also, XLEFC could have potential biotechnological applications. For example, it has been reported that mild dcEF stimulation promotes metabolism in cultured bone cells [56]. EF also promotes metabolism and cell growth of hybridoma cells, yielding higher monoclonal antibody production [57]. High voltage application in electrical stimulation bioreactor with large culture area can also aid large-scale electroporation or electrofusion of cells. XLEFC has the potential to be used in such applications.

### D: The temperature in XLEFC shows little Joule heating after dcEF stimulation

**Figure 7:**
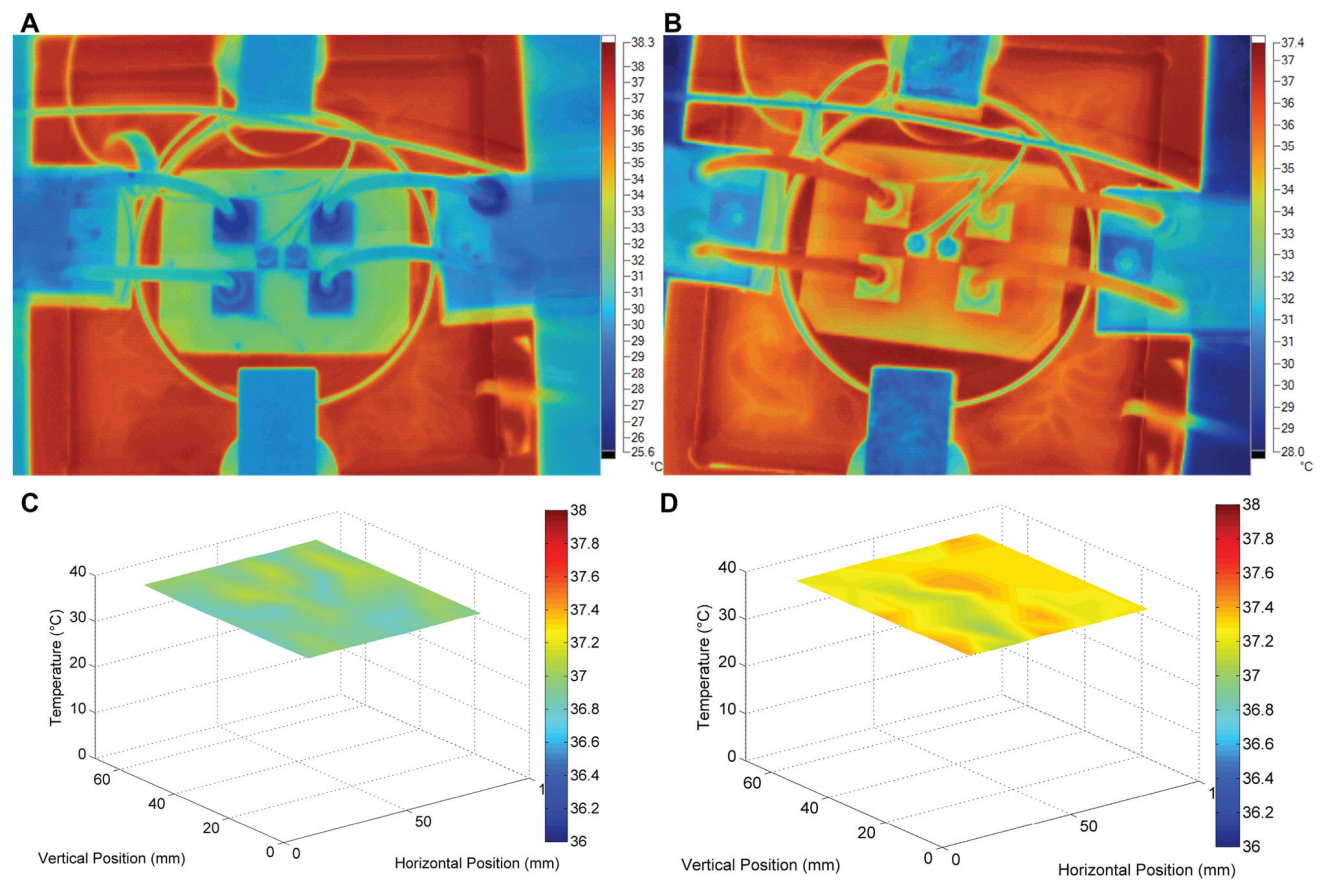
The homogeneous temperature distribution in XLEFC. (A) The infra-red thermo-image of XLEFC before dcEF stimulation. (B) The infra-red thermo-image of XLEFC after 2 hours of dcEF stimulation. (C) The plot of temperature measurement in XLEFC before dcEF stimulation. (D) The plot of temperature measurement in XLEFC after 2 hours of dcEF stimulation.

In addition to confirming the homogenous EF, we also measured the temperature distribution in the culture chamber. The electrical power consumed in the cell culture chamber was about 0.5W (~= 300 mV/mm x 100 mm x 17.14 mA) at the required current of 17.14 mA. Possible Joule heating was speculated. We therefore examine the temperature of XLEFC during the experiment. The infra-red thermo-images of XLEFC before and after dcEF stimulation are shown in Figure 7A and 7B, respectively. The corresponding measured temperature distribution in the chip was plotted as Figure 7C and 7D. The mean measured temperature before the experiment was 36.9° C with CV of 0.2%. After dcEF stimulation, the mean temperature in the cell culture chamber is 37.3° C with CV of 0.2%. The small temperature variation before and after the EF stimulation allows homogeneous cell growth.

**Figure 8:**
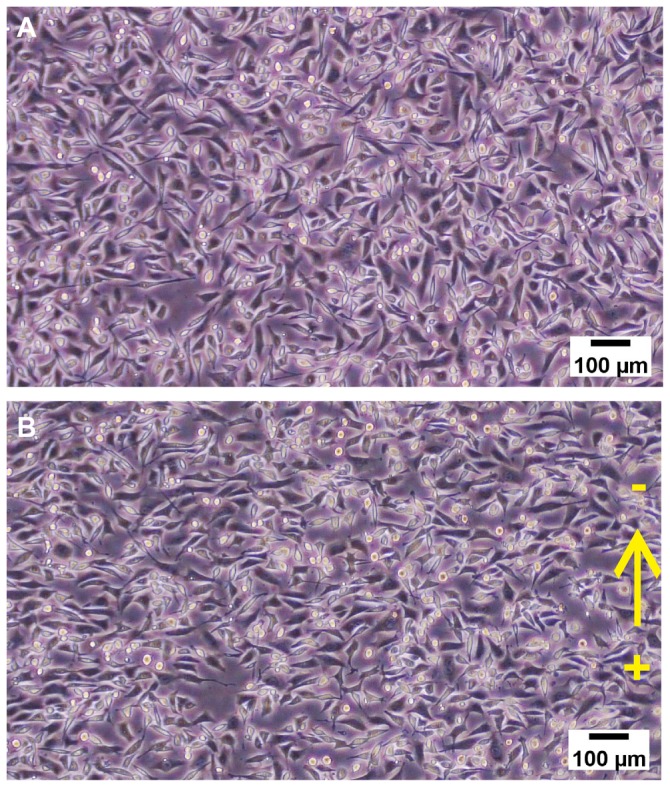
CL 1-5 cells grown in XLEFC. (A) The phase-contrast microphotograph of CL 1-5 cells before dcEF stimulation and (B) after 2 hours of dcEF stimulation. Typical perpendicular electro-alignment was observed. Scale bar: 100µm.

The phase contrast microscope images of CL 1-5 cells before and after 2 hours of dcEF application are shown in Figure 8. CL 1-5 cells demonstrate typical perpendicular electro-alignment against the EF vector [26].

In summary, with homogeneous EF and temperature distribution provided by XLEFC, we can confidently obtain cells that are cultured in a well-controlled environment and can obtain high quality samples for protein analysis.

### E: No RTK phosphorylation in CL1 cells after the dcEF stimulation

The generally accepted hypothesis of electrotaxis mechanism is that dcEF-induced membrane receptor polarization results in directional ligand sensing [2] or initiation of ligand-independent activation [58,59]. The signaling discrepancy initiates polarized intracellular signaling cascades leading to directed cell migration.

RTKs are cell surface receptors that are activated upon recognition of extracellular ligands and are key regulators of extracellular signaling. Aberrant RTK signaling has a pivotal role in the development of various cancers [60,61].

Various RTKs have been reported in the electrotaxis of different types of cells. The EGFR pathway’s involvement in dcEF-cell interaction has been reported in epidermoid carcinoma cell A431 [62], bovine corneal epithelial cell [52,63], human keratinocyte [64,65], A549 lung adenocarcinoma cell [25], and MDA-MB-231 breast cancer cell [24]. Hepatocyte growth factor receptor (HGFR) has been reported to be related to the electrotaxis of bovine corneal epithelial cell [38]. Vascular endothelial growth factor receptor (VEGFR) is known to be involved in dcEF-induced pre-angiogenesis in endothelial cells [37,40]. Involvement of Trk receptors in the dcEF-stimulated growth cone guidance has also been reported [39].

In previous studies [24,39,40], comprehensive investigation of RTK activation was not possible due to the low number of collectable cells. In the present study, taking the advantage of high cell yield of XLEFC, we screened for the RTK activation in CL1 cells. EGFR is of particular interest owing to its high expression and correlation in the electrotaxis of lung and breast cancer cells.

**Figure 9:**
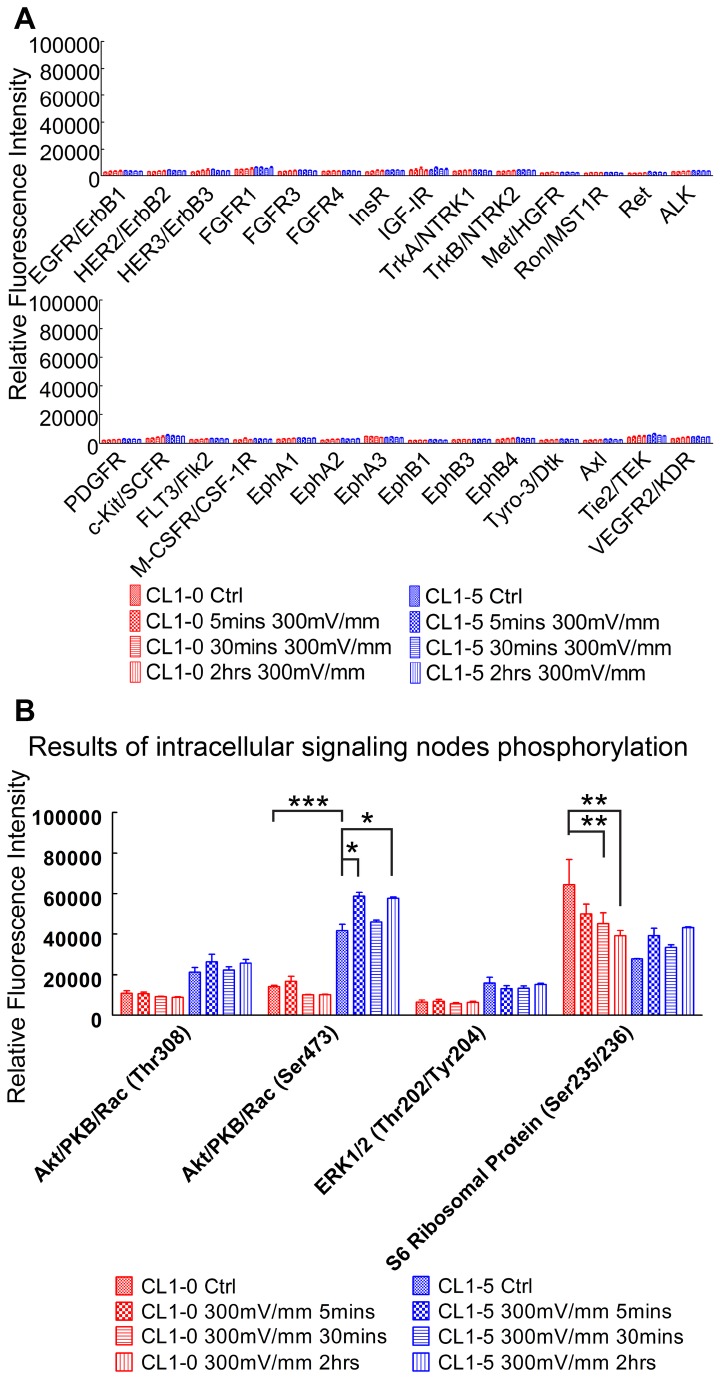
The RTK activation and phosphorylation of CL1 cells determined by the PathScan RTK kit. (A) Bar chart showing the level of RTK activation in CL1 cells with and without dcEF stimulation. No tyrosine phosphorylation of the 28 RTKs was detected in CL1 cells under dcEF stimulation. (B) Bar chart showing the level of phosphorylation of four intracellular signaling nodes in CL1 cells with and without dcEF stimulation. CL1-0 cells show time-dependent phosphorylation decrease in rpS6 while CL 1-5 cells show phosphorylation increase in rpS6 and Akt axis.

**Figure 10:**
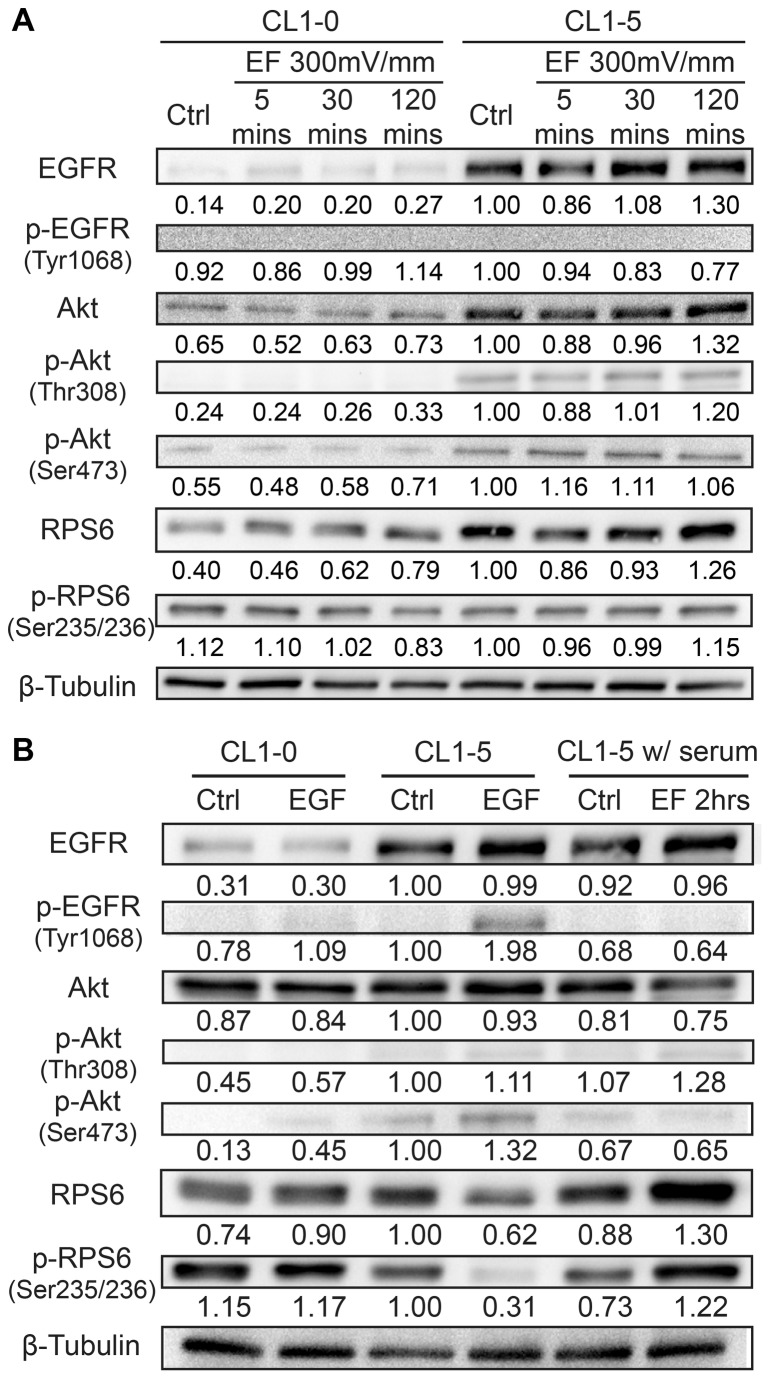
The Western blotting result of CL1 cells under different conditions. (A) Under different period of dcEF stimulation. Note that CL 1-5 cells have high EGFR expression in comparison to CL1-0 cells but neither cells showed EGFR phosphorylation at Tyr1068 under dcEF stimulation. (B) CL1-0 cells and CL 1-5 cells stimulated with 20 ng/mL EGF and CL 1-5 cells stimulated under dcEF in serum-containing medium. Note that even in serum-containing medium, CL 1-5 cells show no EGFR Tyr1068 phosphorylation under dcEF stimulation, contrary to EGF-stimulated cells. The numbers below each protein band indicate the relative densitometry intensity of the protein in different conditions compared to that in the CL 1-5 cells in the control condition.

RTK assay results (Figure 9A) showed that none of the 28 RTKs was phosphorylated at tyrosine residues in both the CL 1-5 and CL1-0 cells under the dcEF stimulation. Absence of EGFR activation in CL 1-5 cells under dcEF was validated by Western blotting against phosphorylated EGFR (Tyr1068), which is shown in Figure 10A. Tyr1068 is a common residue of EGFR that is phosphorylated when EGFR is activated by EGF [66–68].

Despite that the CL 1-5 cells have at least three times higher EGFR expression (Figure 10B) than the CL1-0 cells and that the CL 1-5 cells demonstrate a cathodal distribution of EGFR under dcEF [32], no tyrosine phosphorylation at EGFR or other RTKs were observed when CL1 cells were stimulated in the serum-free medium. A simple explanation for the lack of phosphorylation may be the lack of responsible ligand(s) in the serum-free medium. However, as shown in Figure 10B, the CL 1-5 cells stimulated by a dcEF in the serum-containing medium also did not show tyrosine phosphorylation in EGFR. In contrast, EGF stimulation in CL1 cells did elicit phosphorylation of EGFR (Y1068).

With the fact that the electrotaxis of the CL 1-5 cells is serum-independent, the above results suggest that although CL 1-5 cells has high EGFR expression, and EGFR has been found to participate in the tumorigenesis [69], EGFR is not directly involved in the electrotaxis of the CL 1-5 cells, at least not through conventional ligand-induced EGFR activation. An alternative explanation for this result could be that a dcEF may activate other phosphorylation sites of EGFR. In order to investigate which phosphorylation site(s) is(are) activated under dcEF, further multiple-site screening will be pursued in the future. Again, XLEFC will be very helpful in providing sufficient amount of sample for high-throughput screening.

### F: Distinct intracellular signaling patterns in the electrotaxis of CL1 cells

The PathScan RTK kit also provides phosphorylation information of 11 intracellular biochemical signaling proteins. With this tool, the intracellular signaling patterns in CL1 cells under different durations of dcEF stimulation were investigated. Akt (pThr308), Akt (pSer473) and rpS6 (pSer235/236) were the three phosphorylated proteins with most significant changes in CL1 cells under the dcEF stimulation (Figure 9B).

Akt, also known as protein kinase B, is a serine/threonine kinase acting as a signaling intermediate in numerous cellular processes including proliferation, survival, cell cycle regulation, and differentiation [70,71]. S6 ribosomal protein (rpS6) is a component of the eukaryotic 40S ribosome. Stress or ligand binding signaling has been reported to regulate the phosphorylation of rpS6 through PI3K/Akt/S6K pathway or Ras/MEK/ERK/RSK pathway. Although the biochemical outcome of rpS6 remains unclear, phosphorylation of rpS6 have been shown to regulate global protein synthesis, growth control, glucose homeostasis, and cell size [72–74].

The RTK assay data (Figure 9B) shows that before dcEF stimulation, the phosphorylation profile of intracellular signaling proteins in CL 1-5 cells is different in comparison to that of CL1-0 cells. CL 1-5 cells had higher phosphorylated Akt while CL1-0 cells had higher phosphorylated S6 ribosomal protein (p-rpS6).

After dcEF stimulation, CL 1-5 cells showed slightly increased phosphorylation in Akt (Ser473), which suggests induced signaling in PI3K/Akt by dcEF. This response is similar to what has been reported in electrical signal-controlled wound healing [21]. CL 1-5 cells also show increased rpS6 phosphorylation. Interestingly, dcEF-stimulated CL1-0 cells do not present significant increase in Akt phosphorylation but demonstrate gradual decrease of rpS6 phosphorylation in a time dependent manner. Figure 10A shows that the Western blotting results coincide with the protein phosphorylation dynamics of CL1 cells under the dcEF stimulation observed in the PathScan RTK array.

The discrepancy of Akt and rpS6 phosphorylation dynamics in CL1-0 cells and CL 1-5 cells under dcEF stimulation indicates a fundamentally different response to dcEF in the two types of cells that have different invasivenesses. Electric field has been known to induce cell proliferation and could promote invasion of CL 1-5 cells through activating Akt [75–78]. Although the function of rpS6 in CL1-0 and CL 1-5 cells have not been studied, the contrary response of rpS6 phosphorylation in these two types of cells suggests the need for further investigation into protein homeostasis in cancer cells under dcEF stimulation. The XLEFC developed in this work could be very helpful in expediting such protein level analysis.

### G: EGF-stimulated CL1 cells demonstrate different phosphorylation response compared to dcEF-stimulated CL1 cells

Electrotaxis has been known not exclusively mediated by chemotaxis [79]. As discussed above, the electrotaxis of the CL 1-5 cells was not dependent on EGFR signaling. We further investigated if the CL1 cells respond differently in intracellular signaling under the dcEF stimulus and the EGF stimulus using Western blotting.

Figure 10B shows the Western blotting results of EGF-stimulated CL1 cells and dcEF-stimulated CL 1-5 cells in the serum-containing medium. The CL 1-5 cells stimulated by the dcEF in the serum-containing medium showed similar results compared to those stimulated in the serum-free medium as shown in Figure 10A. Increased Akt and rpS6 phosphorylation were observed in the CL 1-5 cells under the dcEF stimulation in the serum-containing medium, in agreement with the results in the RTK assay described in section F.

EGF stimulated CL 1-5 cells show significant EGFR (Tyr1068) phosphorylation compared to the control group, while neither CL 1-5 cells nor CL1-0 cells show EGFR Tyr1068 phosphorylation under the dcEF stimulation. Both the EGF-stimulated CL 1-5 cells and CL1-0 cells showed slightly increased phosphorylation of Akt (Thr308) and Akt (Ser473). This is in accordance with the EGF activated EGFR/PI3K/Akt signaling for cell proliferation and survival [80].

Similar to the findings in the RTK assay, the Western blotting result shows that the dcEF stimulation and the EGF stimulation on the CL1 cells resulted in different phosphorylation dynamics of rpS6 in the two types of cells. With or without serum in the culture medium, the dcEF-stimulated CL 1-5 cells showed increased rpS6 phosphorylation. However, in EGF-stimulated CL 1-5 cells, decreased rpS6 phosphorylation was observed. In the dcEF-stimulated CL1-0 cells, rpS6 phosphorylation decreased over time while no significant change was observed in the EGF-stimulated CL1-0 cells.

The discrepancy in phosphorylation dynamic under the EGF stimulation and the dcEF stimulation suggests that dcEF stimulation in CL1 cells elicits signaling cascades that are different from the EGFR ligand stimulated cascade. This result supports that EGFR in CL1 cells is not activated by the dcEF stimulation.

## Conclusion

In this study, we investigated the serum dependency and EGFR signaling in the electrotaxis of CL 1-5 cells. We found that the electrotaxis of CL 1-5 cells was serum independent and EGFR independent. Blocking of EGFR signaling by Erbitux has no effect on the electrotaxis of the CL 1-5 cells in spite of the high EGFR expression in the cells.

Protein level analysis was easily carried out with the aid of the new dcEF stimulation device, XLEFC, to collect a large amount of cell lysate. XLEFC has more than six fold increase in cell culture area compared to conventional devices and it also holds several advantages for easy experimental operation. Uniform dcEF as well as temperature distribution and negligible Joule heating are achieved in XLEFC, confirming its usefulness for electrotaxis studies.

A commercial antibody array revealed no RTK activation in both CL1-0 and CL 1-5 cells under the dcEF stimulation. CL1-0 cells and CL 1-5 cells under the dcEF stimulation show different phosphorylation profiles of several intracellular signaling proteins. Akt and rpS6 phosphorylation were increased in the CL 1-5 cells under the dcEF stimulation while in the CL1-0 cells, rpS6 phosphorylation was decreased. This suggests that the two cell lines from the same tissue origin but with different invasiveness respond differently toward the same dcEF stimulation.

The phosphorylation of Akt and rpS6 were also investigated in EGF-stimulated CL1 cells. Both CL1-0 and CL 1-5 cells show increase in Akt phosphorylation under the EGF stimulation. In the EGF-stimulated CL1-0 cells, rpS6 phosphorylation was increased. In contrast, rpS6 phosphorylation in the CL 1-5 cells was decreased following the EGF stimulation. This result was opposite to what was found in the dcEF-stimulated CL1 cells, where rpS6 phosphorylation is decreased in the CL1-0 cells and increased in CL 1-5 cells. The contradicting results further suggest that the cellular signaling cascades involved in the CL1 cells under dcEF are different from those under EGF stimulation and the electrotaxis of the CL 1-5 cells does not involve ligand-induced signaling of EGFR pathway.

As the dcEF stimulation can elicit disparate cellular signaling in different types of cells, physiological electric field could play essential roles in tumorigenesis and cancer heterogeneity. With the aid of the new device developed in the present work, proteomic approach can be carried out in the future to elucidate the cellular signaling network under the dcEF stimulation and identify the molecular mechanism for electrotaxis in different types of cells.
